# Probiotic Effects of *Bacillus licheniformis* DSM5749 on Growth Performance and Intestinal Microecological Balance of Laying Hens

**DOI:** 10.3389/fnut.2022.868093

**Published:** 2022-04-27

**Authors:** Xue Pan, Yuanli Cai, Linglian Kong, Chuanpi Xiao, Qidong Zhu, Zhigang Song

**Affiliations:** ^1^Department of Animal Science and Technology, Shandong Agricultural University, Taian, China; ^2^College of Life Science, Qilu Normal University, Jinan, China; ^3^Precision Livestock and Nutrition Unit, Gembloux Agro-Bio Tech, University of Liège, Gembloux, Belgium

**Keywords:** *Bacillus licheniformis*, production performance, laying hens, intestinal health, microbiota

## Abstract

This study was conducted to investigate the effects of *Bacillus licheniformis* DSM5749 on the production performance and intestinal health in laying hens. A total of 32-week-old laying hens (Hyline Brown) were randomly assigned to two dietary groups (10 replicates of 27 laying hens), namely, basal diet and basal diet complemented with 200 g/t *B. licheniformis* (3.2 × 10^9^ CFU/kg). The trial lasted for 8 weeks, and samples were collected at the last week. Results revealed that *B. licheniformis* DSM5749 significantly improved laying performance, including an increase in egg production rate and average daily egg yield, and a decrease in the feed-to-egg ratio during the entire 8-week experimental period (*P* < 0.05). *B. licheniformis* DSM5749 increased the levels of superoxide dismutase and glutathione peroxidase in the liver and decreased the IL-1 level in the serum (*P* < 0.05). In addition, the integrity of intestinal morphology (villus height, crypt depth, and villus height/crypt depth), tight junctions (*ZO-1*, *Claudin-1*, and Occludin), and lipase vitality in the intestine were potentiated by *B. licheniformis* DSM5749 in laying hens (*P* < 0.05). *B. licheniformis* DSM5749 decreased the Firmicutes/Bacteroidetes ratio (*P* < 0.05) in the cecum. Furthermore, *B. licheniformis* DSM5749 modulated the microbiota in the cecum of the laying hens, increased the relative abundance of beneficial bacteria (e.g., *Prevotella*) at the genus level and decreased the relative abundance of potential pathogens (e.g., *Desulfovibrio*). In conclusion, *B. licheniformis* DSM5749 can improve laying performance, promote intestinal health, affect the composition of cecal microorganisms, and regulate the intestinal micro-ecological balance, making *B. licheniformis* a good probiotic candidate for application in the laying hens industry.

## Introduction

Antibiotics are chemical substances produced by microorganisms that play a remarkable role in disease prevention and growth promotion in the poultry industry. However, residual and resistance problems caused by the long-term use of antibiotics lead to economic losses of livestock and the eventual ban of antibiotic growth promoters, which bring challenges and opportunities to the livestock industry and pressure the industry to find alternatives that are environmentally friendly, green, organic, and efficient for improving poultry growth performance (1). Under these conditions, there is a growing interest in supplementing and/or supporting a natural and beneficial microbiota, which appears to be a promising natural remedy (2, 3). Probiotics, beneficial bacteria that can positively affect the health of poultry (4), can improve growth performance and egg quality (5), ameliorate intestinal morphology and barrier function and enhance the immunoregulatory functionalities of poultry (6).

Recent research revealed that probiotics can promote feed conversion efficiency (7), improve egg production and shell quality (8), and regulate the colonization of symbiotic bacteria (9). *Bacillus* sp. has been reported to indicate promising applications as a probiotic for feeding because of its inherent ability to sporulate and survive under environmental stress. Spore formers manifest tremendous tolerance and survivability in extreme temperatures, pH, dehydration, or malnutrition (10). In addition, *Bacillus* probiotic biofilms incorporate biochemical effects such as antimicrobial and enzymatic activity, thus modulating immune system activity and helping to preserve poultry from the gastrointestinal tract (GIT) and other infections (11). *Bacillus licheniformis* K-508, an aerobic probiotic, possesses the capacity to improve the degradation, absorption, and utilization of nutrients (12), and *B. licheniformis*-fermented products can suppress the growth and multiplication of pathogens to promote intestinal health (13). Research on broilers demonstrated that supplementing diets with *B. licheniformis* HJDY01 can improve growth performance, increase short-chain fatty acid production, and regulate cecal microbiota in broilers (14). Meanwhile, some studies indicated that *B. licheniformis* H2 normalized the ileal microbiota of chickens infected with necrotic enteritis (15) and decreased the incidence of diarrhea in weaning piglets (16). Recent studies have shown that dietary *B. licheniformis* PY79 (17) and *B*. *licheniformis* CGMCC 1.3448 (18) can improve egg production performance and egg quality, ameliorate the adverse influence of heat on egg production (19) and regulate reproductive hormone secretions of laying hens (18).

To our knowledge, comprehensive studies on the intestinal health of *B. licheniformis* DSM5749 are scarce, and no relevant systematic study has been conducted on laying hens. Furthermore, *B. licheniformis* DSM5749 is a novel product, and no publication data are available. Therefore, the present experiment was conducted to investigate the effects of *B. licheniformis* DSM5749 on the laying performance, serum indicators, antioxidant enzyme capacity, and intestinal microecological balance of laying hens to furnish a reference basis for the implementation of *B. licheniformis* DSM5749 in laying hen production.

## Materials and Methods

### Experimental Design and Management

A total of 540 32-week-old laying hens (Hyline Brown) with good condition and similar weight were randomly divided into two groups: basal diet (T1 group) and basal diet supplemented with 200 g/t *B. licheniformis* powders (3.2 × 10^9^ CFU/kg, T2 group). The additive dosage of *B. licheniformis* was optimized according to previous studies (18). Each group contained 10 replicates of 27 laying hens. The ingredient and nutrient levels in the basal diet are shown in Table 1. The laying hens were housed in a fully enclosed chicken house with a one-story ladder cage system and a relative humidity of approximately 65% during the trial period and received water and diet *ad libitum*. Fifteen hours (6:00 to 21:00 h) of artificial lighting with a light intensity of 20 lux was provided daily. The temperature was kept at 20–24°C, and eggs were picked up manually at 14:00 daily. Manure was cleaned regularly, and the hens were immunized according to normal immunization procedures. The experimental *B. licheniformis* (DSM5749) was provided by Chr. Hansen (Beijing) Trading Co., Ltd. (Beijing, China).

**TABLE 1:** Composition and nutrient levels of basal diets (air-dry basis).

Items	Content (%)
**Ingredients**	
Corn	59.00
Rice bran oil	0.80
Breadcrumbs	2.00
Soybean oil	16.90
Corn gluten meal	4.00
DDGS	4.00
Brewing yeast cultures	1.00
Shell powder	7.80
CaHPO4	1.00
Premix^1^	3.50
Total	100
**Nutrient level^2^**	
Metabolizable energy (Kcal/kg)	2650
Crude protein	15.50
Calcium	3.96
Methionine	0.44
Lysine	0.80
Total phosphorus	0.58

*^1^The premix provided the following per kilogram of the diet: vitamin A, 6000 IU; vitamin D_3_, 2500 IU; vitamin B_1_, 1.75 mg; vitamin B_2_, 5.5 mg; vitamin B_6_, 4 mg; vitamin B_12_, 0.18 mg; vitamin E, 25 mg; vitamin K_3_, 2.25 mg; Cu, 7.5 mg; Mn, 60 mg; Fe, 75 mg; Zn, 60 mg; Se, 0.15 mg; biotin, 0.14 mg; NaCl, 3.7 g; folic acid, 0.8 mg; pantothenic acid, 12 mg; phytase, 400 U; nicotinic acid, 34 mg; chloride, 350 mg.*

*^2^Nutrient levels were all calculated values.*

### Laying Performance

Under the same management conditions, eggs were collected daily, and data on total eggs, egg weight, dirty eggs, broken eggs, soft eggs, malformed eggs, and feed consumption were recorded in duplicate. The egg production rate, qualified rate, average egg weight, average daily egg yield, and feed-egg ratio were subsequently calculated.

### Sample Collection

One laying hen per replicate was randomly selected for sampling after laying performance was determined. Blood samples were collected from the wing vein using heparinized anticoagulant tubes. Then, the tubes were shaken gently and transferred to ice. Plasma was obtained after centrifugation at 4,000 rpm for 10 min at 4°C and stored at −20°C for further biochemical analysis. Laying hens were slaughtered by cervical dislocation after blood samples were obtained. Approximately 2 cm segments were excised from the jejunum (from the entry point of the bile duct to Meckel’s diverticulum), flushed repeatedly with cold saline solution, and immediately immersed in 4% paraformaldehyde solution for subsequent histological examination. Tissue samples were obtained from the liver and jejunum. Liver samples at the apical region of the liver were used for further analysis of lipid metabolism and antioxidation capacity. Jejunum samples were collected in the jejunal middle region and divided into two segments, one for gene expression analysis and the other for analysis of digestive enzyme activity and immunity parameters. The samples were washed with ice-cold sterilized saline solution, immediately frozen in liquid nitrogen, and stored at −80°C. The cecum was collected on ice, transported to the laboratory in a dry-ice bag, and then stored at −80°C for 16S rRNA high-throughput sequencing analysis.

### Serum Analysis

Glucose (GLU), triglyceride (TG), total cholesterol (TC), low-density lipoprotein (LDL), and high-density lipoprotein (HDL) were analyzed using a fully automatic biochemical analyzer (7020; Hitachi, Co., Ltd., Tokyo, Japan). The immune response status in the serum was estimated by detecting the levels of interleukin 1 (IL-1) and immunoglobulin G (IgG) with enzyme-linked immunosorbent assay (ELISA) kits (MLBIO Co., Ltd., Shanghai, China). All operations were carried out in accordance with the kits’ instructions. The inter- and intra-assay coefficients of variation (CVs) were less than 10%.

### Liver Analysis

Triglyceride (No. A110-1-1), total cholesterol (No. A111-1-1), and lipase viability (No. A054-1-1) contents in the liver were detected by biochemical methods following the instructions provided with the reagent kits purchased from Nanjing Jiancheng Bioengineering Institute of China. Superoxide dismutase (SOD, No. ml600830) and glutathione peroxidase (GSH-Px, No. ml036972) were estimated using ELISA kits from MLBIO Co., Ltd. (Shanghai, China).

### Jejunal Morphology Analysis

The intestinal samples were trimmed and washed to remove the intestinal contents. The samples were dehydrated and embedded in paraffin following routine procedures. A tissue section was stained with hematoxylin and eosin for histological observation. Jejunal sections were scrutinized using ImageJ analysis software (version 1.47, Bethesda, MD, United States). Villus height (VH) and crypt depth (CD) were measured from 10 random villi in each section. Afterward, the VH-to-CR ratio (VH/CD) was calculated. The final result was expressed as the average of 10 measurements for statistical analysis.

### Biochemical Assay of Jejunum

Jejunum tissue samples were homogenized, and then the homogenates were centrifuged at 4,000 rpm for 20 min at 4°C. Jejunal protease (No. A080-2), lipase (No: A054-1-1), and amylase (No. C016-1-1) activities were determined with diagnostic kits purchased from Nanjing Jiancheng Biotechnology Institute (Nanjing, China), and secreted immunoglobulin A (sIgA) levels were detected with ELISA kits (MLBIO Co., Ltd., Shanghai, China) according to the manufacturer’s instructions. The results were normalized to the protein concentration in each jejunal homogenate.

### RNA Isolation and Quantitative Polymerase Chain Reaction

Gene expression in the jejunum was assessed using quantitative real-time polymerase chain reaction (PCR) with SYBR Green I labeling. Total RNA from the jejunum (*n* = 6) was extracted using TRIzol (Invitrogen Biotechnology Inc., CA, United States) according to the manufacturer’s protocols. Extracted RNA was dissolved in RNase-free water and quantified with a DeNovix spectrophotometer (DS-11, DeNovix Inc., Wilmington, DE, United States). The samples with A260/A280 ratios of 1.8–2.0 and A260/A230 ratios of 2.0–2.2 were chosen for subsequent PCR. RNA integrity was assessed through 1% agarose gel electrophoresis. Reverse transcription of total RNA was performed using the PrimeScript^®^ RT reagent kit with gDNA Eraser (RR047A, Takara Bio Inc., Dalian, China). Real-time PCR was carried out using an Applied Biosystems 7500 Real-time PCR System (Applied Biosystems, Foster, CA, United States). The reaction procedure was as follows: predenaturation at 95°C for 10 s, denaturing by 40 cycles at 95°C for 5 s, annealing, and extension at 60°C for 34 s at the end. The primer sequences are described in Table 2. SYBR green fluorescence was measured at the end of each cycle to monitor the amount of PCR product, and a standard curve was plotted to calculate the efficiency of the real-time PCR primers. The relative expression of target genes was analyzed by the 2^–ΔΔCt^ method (20) after the normalization of the geometric means of β-actin and glyceraldehyde-3-phosphate (GAPDH) expression. Each sample was assayed in triplicate.

**TABLE 2:** Primer sequences for fluorescent quantitative PCR.

Genes	Accession number	Primer sequences (5′→3′)	Product size (bp)
β-actin	NM_205518.1	F: CACCACAGCCGAGAGAGAAA R: CACAGGACTCCATACCCAAGAA	215
*GAPDH*	NM_204305.1	F: GCCCAGAACATCATCCCA R: CGGCAGGTCAGGTCAACA	137
*ZO-1*	XM_015278981.2	F: CTTCAGGTGTTTCTCTTCCTCCTCTC R: CTGTGGTTTCATGGCTGGATC	131
*Claudin-1*	NM_001013611.2	F: CTGATTGCTTCCAACCAG R: CAGGTCAAACAGAGGTACAAG	140
*Occludin*	NM_205128.1	F: GCTCTGCCTCATCTGCTTCTT R: CCCATCCGCCACGTTCTTC	142

*GAPDH, glyceraldehyde-3-phosphate; ZO-1, zonula occludens-1.*

### 16S rRNA Sequencing and Analysis

Samples of the cecal contents were prepared (*n* = 6). Total DNA was extracted from cecal contents with an E.Z.N.A.^®^ Soil DNA Kit (Omega Bio-Tek, Norcross, GA, United States) according to the manufacturer’s instructions. The quantity of extracted DNA was evaluated with a NanoDrop2000 spectrophotometer (Thermo Fisher Scientific, Wilmington, NC, United States), and integrity was examined by 1% agarose gel electrophoresis. Specific barcodes were synthesized based on the V3–V4 regions of the bacterial 16S rRNA gene. The extracted DNA was used as the template for the PCR amplification of the 16S rRNA V3–V4 variable regions.

The universal primers 338F (5′-ACTCCTACGGGAGGCAGCAG-3′) and 806R (5′-GGACTACHVGGGTWTCTAAT-3′) (21) were incorporated into the PCR system. A 20 μL mixture of 10ng template DNA, 0.4 μL of FastPfu polymerase, 0.8 μL of each primer (5 mM), 2 μL of 2.5 mM dNTPs, and 4 μL of 5 × FastPfu buffer was prepared for PCRs with three replicates per sample. The amplification procedure was established with the Applied Biosystems GeneAmp 9700 system (ABI, United States) as follows: 95°C for 3 min, 95°C for 30 s for 27 cycles, 55°C for 30 s, 72°C for 45 s, and 72°C for 10 min. The PCR products were examined by 2% agarose gel electrophoresis, purified using the AxyPrep DNA gel recovery kit (Axygen Biosciences, Union City, CA, United States), and quantified with a QuantiFluor™-ST blue fluorescence quantitative system (Promega, United States). Libraries were constructed, and paired-end reads were sequenced on the Illumina MiSeq PE300 platform (Illumina, San Diego, CA, United States) from Majorbio Biomedical Technologies Ltd. (Shanghai, China).

The raw sequencing data were quality filtered, trimmed, concatenated, and merged according to FLASH (version 1.2.11)^1^ overlap relationships to produce the final quality sequence for subsequent analysis. High-quality sequences with ≥97% similarity were classified by UPARSE (version 7.1)^2^ into identical operational taxonomic units (OTUs). The OTU representative sequence was annotated to species, and the species composition of each sample at different taxonomic levels was counted with the RDP Classifier (version 2.2)^3^ against the Silva 16S rRNA database (release 119)^4^ at a confidence threshold of 70%.

### Data Statistical Analysis

All data were subjected to a T test in SPSS software (SPSS 26.0, SPSS, Chicago, IL, United States). Data were presented as the mean ± standard error (SE). Differences were considered significantly different at *P* < 0.05. A tendency was defined at “0.05 < *P* < 0.1”.

## Results

### Laying Performance

The egg production rate, qualified rate, average daily egg yield, average egg weight, and feed-to-egg ratio were calculated to evaluate the effects of *B. licheniformis* DSM5749 on the laying performance of laying hens (Table 3). After 8 weeks of treatment, the egg production rate and average daily egg yield were increased, whereas the feed-to-egg ratio were decreased in the *B. licheniformis* DSM5749 group compared with those in the control group (*P* < 0.05). Dietary *B. licheniformis* DSM5749 did not affect the qualified rate or average egg weight of laying hens (*P* > 0.05).

**TABLE 3:** Effects of dietary *B. licheniformis* DSM5749 on the production performance of laying hens at 8 weeks.^1^

Items	Egg production rate (%)	Qualified rate (%)	Average egg weight (g)	Average daily egg yield (g/d)	Feed-to-egg ratio (g/g)	Mortality (%)
T1	93.77 ± 0.35^a^	97.99 ± 0.16	60.13 ± 0.10	56.34 ± 0.23^b^	2.01 ± 0.01^a^	0.05 ± 0.04
T2	95.71 ± 0.30^b^	97.78 ± 0.17	60.41 ± 0.09	58.68 ± 0.56^a^	1.96 ± 0.01^b^	0.04 ± 0.04
*P* value	<0.0001	0.374	0.143	<0.0001	<0.0001	0.991

*^1^Data were expressed as the mean ± SE (n = 10).*

*T1: Control Group; T2: B. licheniformis DSM5749 Group.*

*^ab^Different superscripts in the same row indicate significant differences (P < 0.05).*

### Serum Biochemical Indexes and Immune Parameters

We tested the biochemistry profile and immune parameters in the serum of laying hens, including the contents of GLU, TG, TC, HDL, LDL, IL-1, and IgG, to determine the effects of *B. licheniformis* DSM5749 on the health status of laying hens (Table 4). On week 8, *B. licheniformis* DSM5749 reduced the GLU levels (*P* < 0.05) but had no significant influence on the other indicators in the serum (*P* > 0.05). Dietary *B. licheniformis* DSM5749 showed a downregulated level of IL-1 (*P* < 0.05) and a tendency to upregulate IgG levels (*P* = 0.094) in the serum of laying hens (*P* > 0.05).

**TABLE 4:** Effects of dietary *B. licheniformis* DSM5749 on the serum biochemical and immunological indicators of laying hens at 8 weeks.^1^

Items	GLU (mmol/L)	TG (mmol/L)	TC (mmol/L)	HDL (mmol/L)	LDL (mmol/L)	IL-1 (pg/mL)	IgG (mg/mL)
T1	15.35 ± 0.29^a^	17.93 ± 1.80	3.19 ± 0.34	1.21 ± 0.12	0.78 ± 0.06	287.80 ± 10.07^a^	2.32 ± 0.07^A^
T2	14.22 ± 0.20^b^	17.04 ± 1.72	2.84 ± 0.21	1.31 ± 0.11	0.74 ± 0.06	276.49 ± 5.08^b^	2.78 ± 0.25^B^
*P* value	0.004	0.726	0.402	0.547	0.565	0.005	0.094

*GLU, glucose; TG, triglyceride; TC, total cholesterol; HDL, high-density lipoprotein; LDL, low-density lipoprotein; IL-1, interleukin-1; IgG, immunoglobulin G.*

*T1: Control Group; T2: B. licheniformis DSM5749 Group.*

*^1^Data were expressed as the mean ± SE (n = 10).*

*^ab^Different superscripts in the same row indicate significant differences (P < 0.05).*

*^AB^A tendency was defined at “0.05 < P < 0.1”.*

### Hepatic Lipid Metabolism and Antioxidant Indicators

The effects of dietary *B. licheniformis* DSM5749 on liver antioxidant statuses were shown in Table 5. Liver GSH-Px and SOD concentrations were higher in the *B. licheniformis* DSM5749-treated group (*P* < 0.05), whereas TC, TG, and lipase viability in the liver were not affected by dietary *B. licheniformis* DSM5749 (*P* > 0.05).

**TABLE 5:** Effects of dietary *B. licheniformis* DSM5749 on the liver lipid metabolism and antioxidant indicators of laying hens at 8 weeks.^1^

Items	TC (mmol/g prot)	TG (mmol/g prot)	Lipase activity (U/g prot)	SOD (U/mg prot)	GSH-Px (U/g)
T1	0.99 ± 0.35	0.46 ± 0.10	16.07 ± 1.60	705.50 ± 26.69^b^	545.43 ± 41.73^b^
T2	0.78 ± 0.14	0.46 ± 0.08	17.25 ± 2.33	879.33 ± 56.98^a^	779.50 ± 28.85^a^
*P* value	0.596	0.990	0.677	0.024	0.0002

*TC, total cholesterol; TG, triglyceride; SOD, superoxide dismutase; GSH-Px, glutathione peroxidase.*

*T1: Control Group; T2: B. licheniformis DSM5749 Group.*

*^1^Data were expressed as the mean ± SE (n = 10).*

*^ab^Different superscripts in the same row indicate significant differences (P < 0.05).*

### Biochemical Assay of the Jejunum

Digestive enzyme activity, including trypsin activity, lipase viability, amylase activity, and sIgA, were tested to evaluate the digestive enzyme and immunity role of *B. licheniformis* DSM5749 in laying hens (Table 6). *B. licheniformis* DSM5749 improved the viability of jejunum lipase in laying hens (*P* < 0.05) but had no significant influence on the other indicators (*P* > 0.05).

**TABLE 6:** Effects of dietary *B. licheniformis* DSM5749 on intestinal digestive enzyme viability and sIgA of laying hens at 8 weeks.^1^

Items	Trypsin viability (U/mg prot)	Lipase viability (U/g prot)	Amylase viability (U/dL)	sIgA (μ g/mL)
T1	36.12 ± 2.73	16.97 ± 1.82^b^	3.00 ± 0.08	2.80 ± 0.09
T2	37.48 ± 3.19	25.51 ± 1.57^a^	2.66 ± 0.45	2.85 ± 0.10
*P* value	0.548	0.007	0.470	0.678

*sIgA, secretory-type immunoglobulin A.*

*T1: Control Group; T2: B. licheniformis DSM5749 Group.*

*^1^Data were expressed as the mean ± SE (n = 10).*

*^ab^Different superscripts in the same row indicate significant differences (P < 0.05).*

### Jejunal Intestinal Morphology

Crypt depth, VH, and VH/CD were presented in Table 7 to evaluate the effects of *B. licheniformis* DSM5749 on jejunal morphological traits. Intestinal morphology analysis demonstrated that VH exhibited positive responses to *B. licheniformis* DSM5749 (*P* < 0.05). Moreover, CD and VH/CD showed an increasing tendency in laying hens (*P* = 0.090, *P* = 0.056).

**TABLE 7:** Effects of dietary *B. licheniformis* DSM5749 on the intestinal morphology of laying hens at 8 weeks.^1^

Items	VH (μm)	CD (μm)	VH/CD
T1	933.20 ± 1.98^b^	165.40 ± 3.04^A^	5.65 ± 0.11^B^
T2	942.20 ± 1.39^a^	157.20 ± 2.97^B^	6.00 ± 0.12^A^
*P* value	0.005	0.090	0.056

*VH, villus height; CD, crypt depth.*

*T1: Control Group; T2: B. licheniformis DSM5749 Group.*

*^1^Data were expressed as the mean ± SE (n = 10).*

*^ab^Different superscripts in the same row indicate significant differences (P < 0.05).*

*^AB^A tendency was defined at “0.05 < P < 0.1”.*

### Intestinal Gene Expression

**FIGURE 1:**
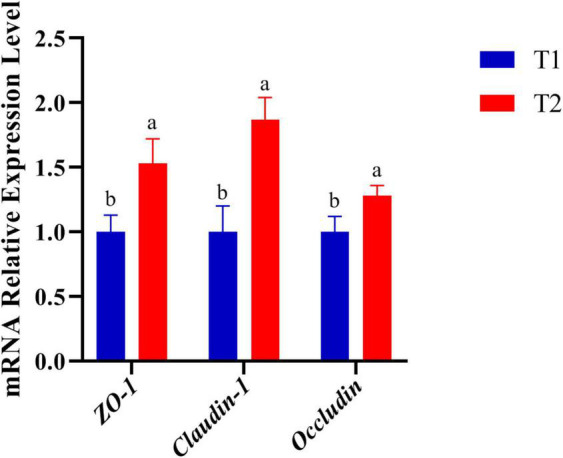
Effects of *B. licheniformis* DSM5749 supplementation on the physical barrier in the jejunum of laying hens. Total RNA was extracted, and the mRNA levels of *ZO*-1, *Occludin*, and Claudin-1 were measured by real-time PCR. *GAPDH*, glyceraldehyde-3-phosphate; *ZO-1*, zonula occludens-1. Different letters indicated values significantly different (*P* < 0.05) among the groups. T1: Control Group; T2: *B. licheniformis* DSM5749 Group.

The expression of genes related to tight junctions was determined in the jejunum by q-PCR to further investigate the molecular basis of the effect of *B. licheniformis* DSM5749 on the intestinal barrier (Figure 1). As shown in Figure 1, the mRNA levels of *ZO*-*1*, Occludin and *Claudin*-*1* were significantly increased in the jejunum (*P* < 0.05).

### Cecal Microbiota Analysis

**FIGURE 2:**
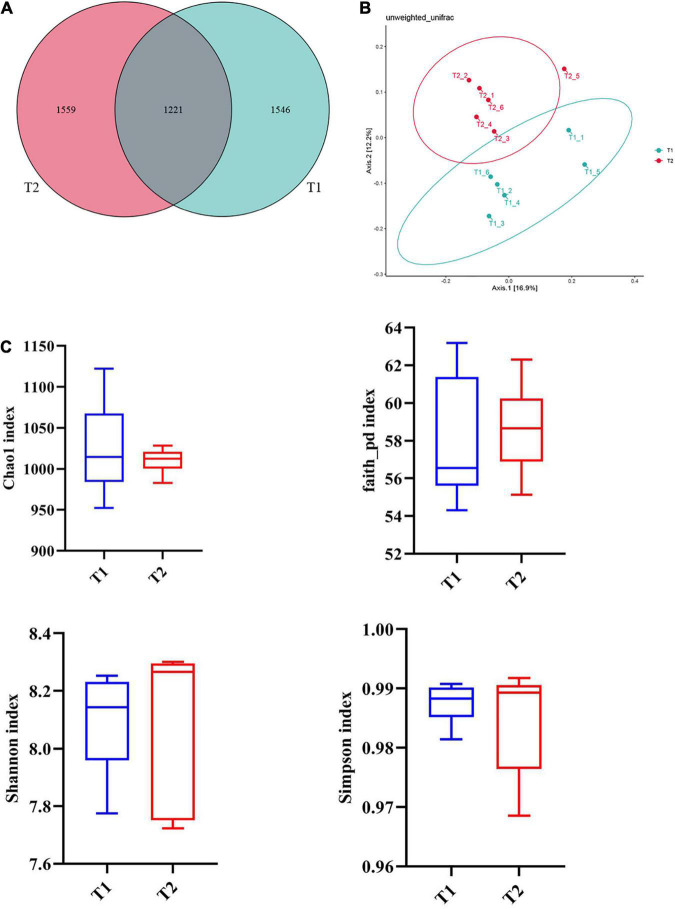
**(A)** Venn diagram between treatments at the OTU level. **(B)** PCoA based on unweighted UniFrac distances. **(C)** An alpha diversity index box graph was established based on the Chao 1, Shannon, Faith_pb, and Simpson indexes. The x-axis represented the group name, and the y-axis represented the alpha diversity index. OTUs, operational taxonomic units. The absence of a superscript on the same row indicated a non-significant difference (*P* > 0.05). T1: Control Group; T2: *B. licheniformis* DSM5749 Group.

**FIGURE 3:**
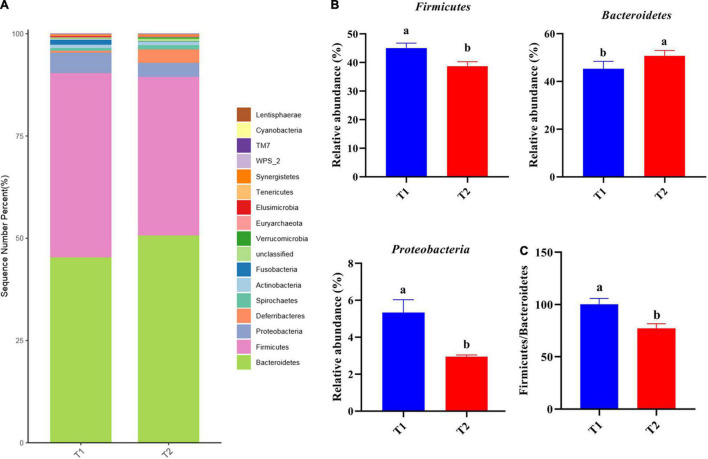
**(A)** The top 20 phyla in the relative abundance of each group. **(B)** Relative abundance of differentially abundant bacteria at the phylum level. **(C)** The ratio of Firmicutes/Bacteroidetes. **(A)** LEfSe analysis of cecal microbiota. Different superscripts indicated significant differences (*P* < 0.05). T1: Control Group; T2: *B. licheniformis* DSM5749 Group.

**FIGURE 4:**
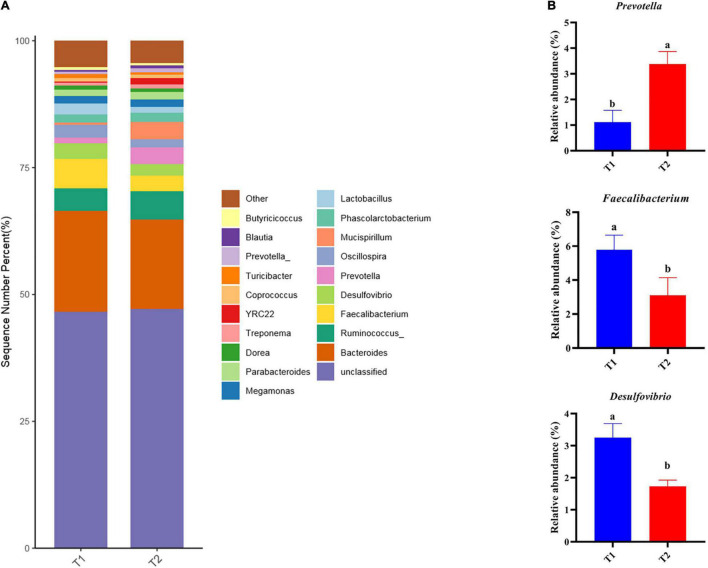
**(A)** Relative abundance of the top 20 bacterial genera were presented in each group, and the relative abundance differed significantly among groups at the genus level. **(B)** The relative abundance differed significantly among groups at the genus level. Different superscripts indicated significant differences (*P* < 0.05). T1: Control Group; T2: *B. licheniformis* DSM5749 Group.

16S rRNA gene high-throughput sequencing was performed to determine the impact of *B. licheniformis* DSM5749 on the cecal microbiota of laying hens. The Venn diagram generated after the OTU clustering of effective tags from all samples with 97% consistency showed that the control group contained 2767 OTUs, and the *B. licheniformis* DSM5749 group contained 2780 OTUs, with 1221 OTUs shared. The control and *B. licheniformis* DSM5749 groups had 1546 and 1559 unique OTUs, respectively (Figure 2A). A principal coordinate analysis (PCoA) was performed to assess similarities and differences among samples and groups (Figure 2B). The *B. licheniformis* DSM5749 group revealed reduced similarity in community structure compared to the control group. The results demonstrated that the *B. licheniformis* DSM5749 diet altered the β diversity index compared to the control diet. *B. licheniformis* DSM5749 had no significant effect on the cecal microbial alpha diversity of laying hens (*P* > 0.05, Figure 2C). Figure 3A showed the changes in the relative abundance of species at the phylum level in the cecum of laying hens. The results indicated that the relative abundances of Firmicutes, Bacteroidetes, and Firmicutes/Bacteroidetes (the ratio of Firmicutes to Bacteroidetes was 1:1) were obviously altered among the control and *B. licheniformis* DSM5749 groups. A reduced proportion of Proteobacteria was observed in the *B. licheniformis* DSM5749 group (*P* < 0.05) (Figure 3B). In addition, the Firmicutes/Bacteroidetes ratio decreased in the ceca of 40-week-old laying hens by *B. licheniformis* DSM5749 (*P* < 0.05) (Figure 3C). Furthermore, the relative abundances of the 20 predominant genera in each group were analyzed to illustrate the specific changes in microbial taxa (Figure 4A). The results showed that *B. licheniformis* DSM5749 enriched the abundance of *Prevotella* (*P* < 0.05) and decreased those of *Faecalibacterium* and *Desulfovibrio* (*P* < 0.05) in week 40 (Figure 4B).

**FIGURE 5:**
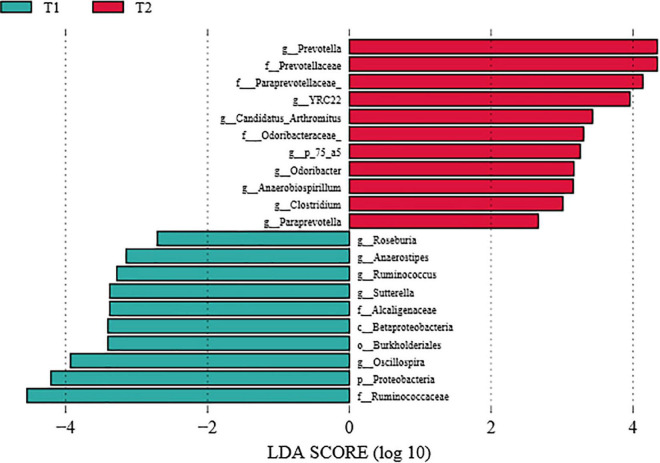
LEfSe analysis of the cecal microbiota of laying hens (LDA score > 4). T1: Control Group; T2: *B. licheniformis* DSM5749 Group.

To further investigate the differences in intestinal flora structure between the different treatment groups, LEfSe analysis was carried out on the cecal contents of the hens, and the results were shown in Figure 5. As seen from the graph, g_*Prevotella* differed significantly between groups and was significantly enriched in the *B. licheniformis* DSM5749 group.

## Discussion

Production performance is closely related to the economic benefits of laying hens. Probiotics, as safe and green microecological agents, has played an important role in maintaining animal health and performance (22). The use of 0.01% 2 × 10^13^ CFU/kg *B. licheniformis* in laying hens was previously found to increase egg production, whereas no significant differences for feed-conversion efficiency (23). In addition, Deng et al. found that 1.0 × 10^10^ CFU/kg *B. licheniformis* could attenuate the negative effects of heat stress on the performance of laying hens (19). Recent studies have shown that the laying rates of hens in 0.01% 1.0 × 10^6^CFU/kg *B. licheniformis* yb-214245 groups were significantly higher than in the control group (24). In the current study, 200g/t 3.2 × 10^9^ CFU/kg *B. licheniformis* DSM5749 increased the egg production rate and average daily egg yield, which was consistent with previous studies (19, 23–25). In contrast, adding 2.0 g/t of a probiotic consisting of *Bacillus subtilis* DSM 32324, *Bacillus subtilis* DSM 32325, and *Bacillus amyloliquefaciens* DSM 25840 had no remarkable effect on egg production, egg weight, or egg yield (26). Feed to egg ratio are important indicators of egg production (27). In the current study, dietary treatment with 200g/t 3.2 × 10^9^ CFU/kg *B. licheniformis* DSM5749 effectively decreased feed to egg ratio of laying hens. Conversely, in the broilers, no significant differences were observed in feed: gain (F:G) ratio with dietary 1.5 × 10^9^ CFU/kg *B. licheniformis* HJDY01 (14). Notably, most research on laying performance has focused on egg production rate and egg quality (19, 23–25). There are scarce reports on the feed-egg ratio we observed in 32-week-old laying hens, which made *B. licheniformis* DSM5749 distinguishable from previous strains. One hypothesis for enhanced laying performance is related to the production of beneficial metabolites by *B. licheniformis*, such as extracellular digestive enzymes, lysozyme, antifungal proteins and various antibiotics (14). Increased levels of lipase viability after *B. licheniformis* DSM5749 treatment may be one of the reasons for the increased laying performance in the present study. In addition, one promising approach is microbial modulation, in which intestinal flora are exploited to enhance laying hens’ immune response to improve production performance (26).

Blood sugar is the main energy supply material of the body and determines the energy supply state of the body and the normal metabolic level of the body. Probiotic preparations can decompose starch and polysaccharides and convert them into monosaccharides, which play a certain role in blood glucose regulation. 1.0 × 10^6^CFU/mL *Lactobacillus rhamnosus* IMC 501^®^ modulates the expression of genes involved in glucose metabolism and reduces whole organism glucose levels in zebrafish larvae (28). The level of serum glucose were lower in hens fed with 1.0 × 10^12^CFU/kg probiotic product called PrimaLac^®^ diet (29) and 2.5 × 10^7^ CFU/kg *Clostridium butyricum* diet (30) compared to those in hens fed with the control diet. In the current study, reduced blood glucose levels were observed in the sera after dietary treatment with 200g/t 3.2 × 10^9^ CFU/kg *B. licheniformis* DSM5749, which is consistent with previous studies (28–31). One potential mechanism may be due to the decomposition of glycogen or gluconeogenesis in laying hens promoted by probiotics (32). Furthermore, another possible reason for this result is that certain probiotics facilitate the production of short-chain fatty acids (SCFAs), leading to the secretion of incretin hormones, which may influence glucose levels (33). The mechanisms of probiotic regulation of blood glucose are currently unclear, and more human interventions and dynamic measurements of insulin sensitivity are needed. It is also necessary to investigate the treatment effects of specific strain(s) at different durations on insulin resistance.

Immunoglobulin G, a major serum glycoprotein acting as an antibody in the immune system, is a principal component of serum immunoglobulin with the longest residence time *in vivo*. This multifunctional sugar interacts with various binding proteins through antigen recognition (34). IL-1, as the predominant inflammatory cytokine, mediates many local and systemic features of inflammation and activates IL-1β, which plays a critical role in the occurrence and proliferation of the host response to invasion and is an essential component in triggering an inflammatory response and provoking immune function (35). In the present study, we found that 200g/t 3.2 × 10^9^ CFU/kg dietary *B. licheniformis* DSM5749 reduced serum IL-1 and showed a gradual increase in IgG levels. Proinflammatory cytokine IL-1 levels decreased in the sera after 5.0 × 10^6^ CFU/kg probiotic treatment with *Lactobacillus acidophilus* LA5 and *Bifidobacterium animalis* subsp. *lactis* BB12 supplementation, which alleviates the systemic inflammatory response in postinfectious (Pi)-irritable bowel syndrome (IBS)-induced mice (36). Moreover, 0.03% *B. licheniformis* CGMCC 1.3448 administration significantly elevates IgG concentrations but drops the secretion of IL-1β (18). A similar result was observed in this study. The results indicated that 200g/t 3.2 × 10^9^ CFU/kg *B. licheniformis* DSM5749 was capable of modulating immune function in laying hens. The cell barrier of *Bacillus* is composed of dextran, which serves as an immune stimulant (37). In addition, previous studies on probiotics have shown that serum IgG, IgM, and IgA showed increased and diversified status in broilers and mice by 1.0 × 10^9^CFU/day *Bacillus subtilis* BS1 or 4.0 × 10^9^CFU/m^2^ *Bacillus amyloliquefaciens*, altering the immune response (38, 39). All these results demonstrated that *Bacillus* has a positive impact on improving the immune capacity. The immunomodulatory effect of probiotics is attributed to the release of cytokines, including ILs, transforming growth factor (TGF), and interferons (IFNs) (40). Furthermore, it has been established that probiotics increase gut barrier functions by influencing cytokine production (41). However, the effects of probiotics on the immunomodulation of cytokines are strain-specific (41). Therefore, in this study, the immunomodulatory effect of *B. licheniformis* DSM5749 on laying hens was reflected in the up-regulation of IgG levels and down-regulation of IL-1.

Superoxide dismutase and GSH-Px, as two important antioxidant enzymes in animal organisms, promote antioxidative stress. GSH-Px, a ubiquitous present *in vivo*, has the capabilities to regulate cellular redox, whereas SOD efficiently converts superoxide anions into hydrogen peroxide, which are degraded to water by GSH-Px (42). A study (23) demonstrated that supplementation with *B. licheniformis* has no impact on improving the antioxidant enzyme activities in laying hens except for glutathione S-transferase (GST). Notably, the present experiment indicated that dietary treatment with *B. licheniformis* DSM5749 effectively improved the GSH-Px and SOD capacity in the liver, suggesting that *B. licheniformis* DSM5749 can decrease lipid peroxidation and enhance the antioxidant capacities of laying hens. These features are beneficial to growth performance (43). Similarly, recent research of broiler chickens found that *B. licheniformis* HJDY01 increased SOD and GSH-Px activity in the serum (14). Previous studies have summarized that probiotics may regulate the redox status of the host through their metal ion chelating ability, antioxidant enzymes, regulatory signaling pathways, and gut microbiota (44, 45). Among these pathways, antioxidant enzymes (SOD, MnSOD, CAT, GSH, and GSH-Px) seem to be the first line of defense during oxidative stress and have a beneficial impact on preventing oxidative damage in poultry (46). This study confirmed the antioxidant effect of *B. licheniformis* DSM5749 on laying hens, suggesting that *B. licheniformis* DSM5749 has potential as a probiotic antioxidant.

The digestive enzymes of poultry consist of protease, amylase, and lipase. Research in broilers found that the activity of digestive enzymes was increased in broilers by supplementation with probiotic *B. coagulans* NJ0516 (47), which was similarly observed in the present study. In addition, Yang et al. (48) reported that compound-supplemented *B. subtilis* yb-114,246 and *B. licheniformis* yb-214,245 improved the activities of chymotrypsin, lipase, and amylase in the digestion of chicken small intestines. The multienzyme digestive system of the intestine provides an environment that promotes nutrient absorption after the administration of *B. licheniformis.* Such a view explains the results of the present study. The data suggested that *B. licheniformis* DSM5749 has a lipase production role, making nutrients more digestible and easily absorbed by the intestine (49).

Small intestinal VH and CD are the key factors for determining nutrient digestion and absorption. One study observed an increase in the VH/CD of laying hens fed a diet supplemented with probiotics, which improved nutrient absorption efficiency and laying production (37). The data indicated that *B. licheniformis* DSM5749 progressively decreased CD while increasing VH and VH/CD of laying hens. The acceleration of VH/CD in the jejunum in the present study supported the idea that *B. licheniformis* mediates laying production through a competitive space mechanism associated with the attenuation of intestinal pathogenic bacteria (19). *B. licheniformis* H2 with anti-inflammatory effects restores the damaged intestinal morphology attacked by subclinical necrotic enteritis (SNE) (45, 50). Therefore, the improvement in intestinal integrity can be partially explained by the decreased expression of the proinflammatory cytokine IL-1 in this study. The intestine maintains relatively high integrity of the tight junction barrier during the normal physiological state of the animal organism, and the transport of toxic intraluminal substances and molecules through tight junctions is well regulated (51). Recent reports have demonstrated that *B. licheniformis* CGMCC 1.3448 enhances epithelial barrier integrity in laying hens (18) and *B. licheniformis* B26 in broiler chickens (52), characterized by the increased expression of *ZO*-1, Occludin and *Claudin-1* at the mRNA level in the gut. A study suggested that *B. licheniformis* preadministration alleviated intestinal injury and improved gut barrier function during and after heat stroke (HS) onset (53). This research indicated that *B. licheniformis* maintained the mucosal barrier and intestinal health. Similarly, in this study, dietary *B. licheniformis* DSM5749 effectively upregulated *ZO-1*, Occludin, and *Claudin-1* expression, demonstrating the beneficial effect of *B. licheniformis* DSM5749 on the intestinal barrier of laying hens.

The balance of microflora in the chicken gut has physiological importance to host health and performance (54). The cecum is the predominant location for microbial fermentation and is the most sought-after part of the chicken gut for research. In this study, the impact of *B. licheniformis* DSM5749 on the intestinal health of laying hens was investigated by analyzing changes in the microbial composition of the cecum at the phylum and genus levels based on species annotation. At the phylum level, Firmicutes and Bacteroidetes accounted for the largest proportion (>80%) of the total microbial community in the ceca of laying hens, which was consistent with previous findings (55). Proteobacteria are often overrepresented in metabolic disorders and inflammatory bowel disease, mostly with an inflammatory phenotype (55). A common trait of Proteobacteria is Gram-negative staining and, thus, the presence of lipopolysaccharide in the outer membrane (56). Carvalho et al. found that mice progressing to colitis showed a definite microbiota signature characterized by increased levels of Proteobacteria, especially of the *Escherichia genus* (57). The present study demonstrated that the cecal microbiota in *B. licheniformis* DSM5749-treated laying hens had a reduced relative abundance of Proteobacteria, suggesting that *B. licheniformis* DSM5749 may have the potential to reduce the incidence of inflammatory diseases in laying hens. This result supports a reduction in serum IL-1 levels in laying hens. An increase in the Firmicutes/Bacteroidetes ratio is the pattern of an impaired intestinal barrier. This leads to the stimulation of regulatory T cells, transport of carbohydrates, and bacterial chemotaxis (58). In the experiment, *B. licheniformis* DSM5749 showed a decreased Firmicutes/Bacteroidetes ratio in laying hens, which may be the potential reason for the enhanced intestinal barrier. To our knowledge, the decreased level of Firmicutes/Bacteroidetes ratio regulated by *B licheniformis* in laying hens’ cecum has been the first-time reported. In addition, the Firmicutes/Bacteroidetes ratio is positively associated with fat storage and is remarkably higher in obese individuals than in adults with normal weight (59). This result may be due to the strong fermentation capacity of Firmicutes, which is more suitable as an energy source than Bacteroides and can produce more short-chain fatty acids and metabolic lipids that contribute to efficient heat absorption and subsequent weight gain (60). In terms of the relative abundance of species at the genus level, *B. licheniformis* DSM5749 increased the amount of *Prevotella*, which contributes to the fermentation of indigestible carbohydrates to butyrate, protein decomposition, and carbon hydration and is capable of promoting the repertoire of liver glycogen in mice (6). This finding indicates that *B. licheniformis* DSM5749 can elevate the decomposition of macromolecular substances, such as protein, in laying hens and accelerate nutrient absorption. There is increasing interest in *Faecalibacterium*, one of the most abundant bacterial species found in the gut, given its potentially important role in promoting gut health. The most notable species in the *Faecalibacterium* genus is *Faecalibacterium prausnitzii* (*F. prau*). There is evidence that the A2-165 strain of *F. prau* has been found to induce a distinct cytokine response, with high IL-10 secretion compared to other *F. prau* strains tested, which is partially explained by higher butyrate production (61). Moreover, experiments on Caco-2 cells showed protective effects at 2 mM butyrate and detrimental effects at 8 mM butyrate (62). Therefore, *F. prau*, a highly abundant butyrate-producing bacterium, has been proposed both as a biomarker for the development of different gut pathologies and as a potential treatment due to its production of anti-inflammatory metabolites; however, studies on forms of administration, and mechanisms of action are still necessary to improve our understanding of the most appropriate use of this bacterium (63). In the present study, the relative abundance of *Faecalibacterium* was lower in the *B. licheniformis* DSM5749 group, which was consistent with previous findings (16). It has been reported that the abundance of *F. prausnitzii* is positively correlated with fasting glucose levels (64), which may be the potential reason for decreased blood glucose levels. A prominent peculiarity of *Desulfovibrio* has been reported is the production of hydrogen sulfide (H_2_S), which is anomerized for sulfate reduction using sulfate as the electron acceptor for respiration (65). H_2_S affects cell signaling in neuronal cells at low concentrations but causes severe toxicities at high concentrations. In addition, *Desulfovibrio* is positively correlated with the amount of H_2_S, which promotes intestinal health by inhibiting the oxidation of short-chain fatty acids, such as butyrate, and disrupting the H_2_S detoxification pathway in intestinal epithelial cells (66). In a recent study of *B. licheniformis* HJDY01 in broiler, no significant differences in the relative abundance of *Desulfovibrio* were demonstrated (14). Interestingly, in this study, the cecal microbiota in the *B. licheniformis* DSM5749-treated laying hens presented a decreased level of *Desulfovibrio*, which means that dietary *B. licheniformis* DSM5749 can maintain intestinal health by reducing the colonization of harmful bacteria.

## Conclusion

In summary, under the conditions of this experiment, dietary *B. licheniformis* DSM5749 has growth-promoting, antioxidant, and anti-inflammatory properties, strengthens the physical barrier function of the intestine by exhibiting a higher expression of the tight junction protein, and induces a healthier microbiota composition characterized by a higher ratio of beneficial bacteria and a lower ratio of harmful bacteria. Therefore, our study demonstrated that *B. licheniformis* DSM5749 could be a potential alternative to antibiotics to enhance the growth performance and maintain the intestinal microecological balance of laying hens, further expanding the resources of strains of laying hen probiotics.

## Data Availability Statement

The datasets presented in this study can be found in online repositories. The names of the repository/repositories and accession number(s) can be found here: https://www.ncbi.nlm.nih.gov/bioproject/797943.

## Ethics Statement

The animal study was reviewed and approved by Ethics Committee of the Shandong Agricultural University.

## Author Contributions

XP designed the experiments and wrote the manuscript. LK performed the *in vivo* experiments. XP, LK, and QZ collected the sample and data. YC analyzed the data. ZS reviewed and modified the final manuscript. All authors read and approved the final manuscript.

## Conflict of Interest

The authors declare that the research was conducted in the absence of any commercial or financial relationships that could be construed as a potential conflict of interest.

## Publisher’s Note

All claims expressed in this article are solely those of the authors and do not necessarily represent those of their affiliated organizations, or those of the publisher, the editors and the reviewers. Any product that may be evaluated in this article, or claim that may be made by its manufacturer, is not guaranteed or endorsed by the publisher.

## Footnotes

1
https://ccb.jhu.edu/software/FLASH/index.shtml

2
http://www.drive5.com/uparse/

3
http://sourceforge.net/projects/rdp-classifier/

4
http://www.arb-silva.de
